# Exploring Usage of COVID Coach, a Public Mental Health App Designed for the COVID-19 Pandemic: Evaluation of Analytics Data

**DOI:** 10.2196/26559

**Published:** 2021-03-01

**Authors:** Beth K Jaworski, Katherine Taylor, Kelly M Ramsey, Adrienne Heinz, Sarah Steinmetz, Ian Pagano, Giovanni Moraja, Jason E Owen

**Affiliations:** 1 National Center for PTSD, Dissemination & Training Division US Department of Veterans Affairs Menlo Park, CA United States; 2 School of Medicine Stanford University Stanford, CA United States; 3 University of Hawaii Cancer Center Honolulu, HI United States; 4 Vertical Design, LLC Berkeley, CA United States

**Keywords:** COVID-19, coronavirus, mobile app, mHealth, digital health, mental health, public mental health, stress, coping, public health, app

## Abstract

**Background:**

The COVID-19 pandemic has significantly impacted mental health and well-being. Mobile mental health apps can be scalable and useful tools in large-scale disaster responses and are particularly promising for reaching vulnerable populations. COVID Coach is a free, evidence-informed mobile app designed specifically to provide tools and resources for addressing COVID-19–related stress.

**Objective:**

The purpose of this study was to characterize the overall usage of COVID Coach, explore retention and return usage, and assess whether the app was reaching individuals who may benefit from mental health resources.

**Methods:**

Anonymous usage data collected from COVID Coach between May 1, 2020, through October 31, 2020, were extracted and analyzed for this study. The sample included 49,287 unique user codes and 3,368,931 in-app events.

**Results:**

Usage of interactive tools for coping and stress management comprised the majority of key app events (n=325,691, 70.4%), and the majority of app users tried a tool for managing stress (n=28,009, 58.8%). COVID Coach was utilized for ≤3 days by 80.9% (n=34,611) of the sample whose first day of app use occurred within the 6-month observation window. Usage of the key content in COVID Coach predicted returning to the app for a second day. Among those who tried at least one coping tool on their first day of app use, 57.2% (n=11,444) returned for a second visit; whereas only 46.3% (n=10,546) of those who did not try a tool returned (*P*<.001). Symptoms of anxiety, depression, and posttraumatic stress disorder (PTSD) were prevalent among app users. For example, among app users who completed an anxiety assessment on their first day of app use (n=4870, 11.4% of users), 55.1% (n=2680) reported levels of anxiety that were moderate to severe, and 29.9% (n=1455) of scores fell into the severe symptom range. On average, those with moderate levels of depression on their first day of app use returned to the app for a greater number of days (mean 3.72 days) than those with minimal symptoms (mean 3.08 days; *t*_1_=3.01, *P*=.003). Individuals with significant PTSD symptoms on their first day of app use utilized the app for a significantly greater number of days (mean 3.79 days) than those with fewer symptoms (mean 3.13 days; *t*_1_=2.29, *P*=.02).

**Conclusions:**

As the mental health impacts of the pandemic continue to be widespread and increasing, digital health resources, such as apps like COVID Coach, are a scalable way to provide evidence-informed tools and resources. Future research is needed to better understand for whom and under what conditions the app is most helpful and how to increase and sustain engagement.

## Introduction

### Impact of COVID-19 on Mental Health and Well-Being

In the United States, the COVID-19 pandemic has led to over 500,000 deaths, millions of job losses, and disruption of nearly every aspect of daily life. COVID-19 has also negatively impacted mental health and well-being globally [1-3]. One-third of American adults report a high level of psychological distress due to the pandemic [4].

Several studies now indicate that an unprecedented mental health crisis is underway. In a poll conducted by Harris [5] on behalf of the American Psychological Association, nearly 8 in 10 adults said the pandemic is a significant source of stress in their lives. The prevalence of depression symptoms among adults in the United States has risen from 8.5% of the population prior to the COVID-19 pandemic to 27.8% in the midst of the pandemic [6]. Researchers from the US Centers for Disease Control and Prevention found that 40% of respondents of a survey administered in June 2020 endorsed at least one adverse mental or behavioral health condition including symptoms of depression, anxiety, posttraumatic stress, or having started or increased substance use to cope with stress or emotions related to COVID-19. Over 10% of respondents reported seriously considering suicide in the previous 30 days [7]. Furthermore, there appears to be a bidirectional relationship between COVID-19 and psychiatric disorders, such that having a psychiatric disorder is associated with a greater likelihood of contracting COVID-19, and contracting COVID-19 is associated with an increased risk of receiving a psychiatric diagnosis [8].

### Digital Mental Health as a Strategy for Addressing the Mental Health Impact of COVID-19

Digital mental health options are needed to help address the mental health effects of COVID-19 as well as the secondary impacts of the pandemic, such as fear of contracting the virus, financial stress related to job loss, loss of childcare, or the need to balance work with remote education. Mobile mental health apps are a promising strategy for addressing mental health impacts of the pandemic because of their potential scalability, reach, and utility, particularly during a time when in-person care may not be accessible due to social distancing and safety regulations. High-quality, accessible, and sustainable apps have been identified as part of an integrated “blueprint” for digital mental health services during the pandemic [9]. They may be a particularly useful tool for reaching a large number of individuals from highly impacted populations at risk for posttraumatic stress disorder (PTSD) or other mental health conditions, including those who have contracted COVID-19 and frontline health care workers [10].

Apps are a particularly appealing medium because of their potential reach. Individuals rarely turn off mobile devices [11], making apps available 24/7. Additionally, in the United States, 81% of adults own smartphones, with few differences among sociodemographic groups [12]. This reach is important because the pandemic has a disproportionate and complex impact on Black, Indigenous, people of color (BIPOC), people from low-income backgrounds, and women [13], and it is clear that vulnerable groups are at greater risk for behavioral and mental health consequences [6,7,14]. Systemic disadvantage with respect to social determinants of health, such as lack of internet access and reduced educational opportunities, has been associated with increased COVID-19 mortality rates [15]. Free, evidence-informed apps, such as COVID Coach, that are developed by government or not-for-profit entities and made specifically to address such systemic barriers, can contribute to a digital mental health safety net for vulnerable individuals. Beyond the ability to reach many people, apps have been shown to be useful adjunctive resources for a range of mental health concerns, including anxiety and depression [16] and PTSD [17].

### Creation of the COVID Coach App

In response to the anticipated mental health impact of the COVID-19 pandemic, and as part of the Veterans Affairs’ (VA) “Fourth Mission” to help during times of national emergencies and support public health, the National Center for PTSD created COVID Coach (Multimedia Appendix 1). COVID Coach is a free, publicly available mental health app designed to help people cope with stress, find resources, and track mental health over time. It is intended to be simple to use, does not require an internet connection or data plan to access primary content, and all recommended activities and resources are low in cost or free to users. COVID Coach is one of only a few public mental health apps available for specifically addressing mental health concerns stemming from or exacerbated by COVID-19, and it is the newest in a suite of free mental health apps designed to support mental health [18,19].

COVID Coach is based upon the model of the empirically supported PTSD Coach app [20], which has been identified as a potential approach for the behavioral and mental health impact of COVID-19 [21]. COVID Coach provides app users with many of the features of PTSD Coach, including tools for coping with challenging situations and managing stress, psychoeducation, tracking of mental health symptoms, and quick access to support networks and crisis resources. COVID Coach also provides symptom management tools adapted for life during the pandemic (eg, sleep struggles; isolation; stress; sadness; and indoor, socially distanced activities), goal-setting, and over 50 unique psychoeducational topics about managing COVID-19–related concerns (ie, staying well, staying balanced, staying together, staying safe, and staying healthy). The app was released at the end of April 2020 and has been promoted as part of the VA’s response to the pandemic and highlighted as an important resource [22].

### Evaluating COVID Coach in the Context of a Public Health Disaster

Mobile mental health apps can be useful tools in large-scale disaster responses [23], and their use has been indicated specifically within the context of the COVID-19 pandemic (eg, [24,25]). However, the utility of standalone apps “in the wild” can be limited by poor engagement and high attrition (eg, [26,27].) A host of challenges renders it difficult to conduct formal research and evaluation on disaster mental health interventions and resources [28]. Accordingly, there is often insufficient data on when, how, and why individuals utilize disaster mental health resources to help guide policy and budgetary allocation. Although COVID Coach has been well received in the general population, usage of the app, particularly the key content areas, and retention have not yet been formally evaluated.

### Objective

This study utilized anonymous mobile analytics data to characterize the overall usage of an app designed specifically to provide tools and resources for addressing COVID-19–related stress, explore retention and return usage, and assess whether the app was reaching individuals that may benefit from mental health resources. Three key aims guided the study: (1) describe general usage trends between May 1, 2020, and October 31, 2020 (a key period of time during the pandemic), and identify how frequently specific types of key app content were used (ie, coping tools, psychoeducation, self-assessments, and accessing resources); (2) explore usage patterns, with a particular focus on understanding how usage of key content on the first day of use may be related to return use and retention; and (3) characterize baseline mental health and well-being among COVID Coach users.

## Methods

### COVID Coach Mobile App Description

COVID Coach, available for Android [29] and iOS [30], is an app designed specifically for the COVID-19 pandemic to provide users with interactive, evidence-informed tools for coping with stress and anxiety, information about how to stay well, stay connected, and navigate challenges, self-monitoring mental health symptoms and goals, and resources to discover and connect with various types of verified and vetted support. The app can be used independently or in conjunction with professional mental health care but is not a replacement for therapy. Users are not required to create an account or log in to access any of the content, and the app is fully compatible with assistive software technologies (eg, VoiceOver or TalkBack).

### Mobile Analytics Data

COVID Coach collects anonymous information about app use for the purposes of quality improvement. Fully nonidentifying, anonymous, and encrypted event sequences were stored using JavaScript Object Notation (JSON) format on a remote GovCloud server that meets VA security and privacy requirements. Data are accessible from VA App Connect software, which has been approved for use under the VA’s Technical Reference Model [31]. Upon first launch of the app, a unique, randomly generated 32-character (256-bit) code is assigned to that particular app installation. Completely anonymous usage data, such as screens selected, button presses, and other nonidentifying patterns, are collected and associated with this install code. Install codes serve as a proxy for app users since the unique identity of each app user cannot be determined. Each in-app event contains a timestamp (in Coordinated Universal Time [UTC]) that corresponds to when the event actually occurred, but data are only transmitted to the server when the app is in use and connected to Wi-Fi or utilizing a data plan.

### Procedures

For the purpose of this study, mobile analytics data with timestamps between May 1, 2020, and October 31, 2020, were extracted from the research server on November 4, 2020. Between May 1 and October 31, 3,368,931 in-app related events were captured (Android: n=847,260; iOS: n=2,521,612) across 49,297 unique install codes (Android: n=12,938; iOS: n=36,359). 

### Measures

#### App Use Metrics

Daily active users and monthly active users were measured by the total number of app users that used COVID Coach on a given day or at least once within a given month. Overall, frequencies for key content usage were computed for each of the four key sections in the app: *Manage Stress* (tried a tool), *Learn* (viewed a learn topic), *Mood Check* (created and rated a goal or completed an assessment), and *Find Resources* (viewed at least one specific subsection within *Find Resources*). These frequencies were computed for all key events and for all app users that had activity during the observation window (May 1, 2020, through October 31, 2020). Based on a rationale similar to Kwasny and colleagues [32], we decided a priori that frequency of use within the observation window would be measured in terms of unique days of use, rather than sessions or visits because of the variability in establishing the end of an app session, within and across platforms. Additionally, all app users were categorized according to whether their first day of app use occurred during the observation window (first-time users) or prior to the start of the observation window. Thus, all analyses related to distinct days of app use, return usage, and patterns of usage by day of use focused only on app events associated with first-time users. Among all first-time users, distinct days of app use within the observation window were calculated, as well as retention days (the number of days between the first day of use and the last day of use) and the number of days between the first day of use and the second day of use (for all individuals who used the app for at least 2 distinct days). For each first-time user, completion of tasks within each of the four key content areas were totaled, by each distinct day of use. First-time users who completed one or more assessments on their first day of app use were identified as “baseline” assessment completers.

#### In-App Assessments

Four assessments are available within the *Mood Check* section of COVID Coach. These assessments can be accessed and taken at any time by app users.

The Warwick-Edinburgh Mental Well-Being Scale (WEMWBS) [33] is a measure to assess the feelings and functional aspects of positive mental health. COVID Coach contains the 14-item version of the scale, with each item measured on a Likert-type scale ranging from 1 (“none of the time”) to 5 (“all of the time”). For each item, respondents are asked to consider how they have been feeling over the past 2 weeks. Total score is obtained by summing all items. Scores of less than 42 are indicative of low well-being [34]. The scale was found to be a valid and reliable tool for measuring mental well-being in diverse populations and across project types, and has adequate internal reliability (α=.89) [35].

The Generalized Anxiety Disorder-7 (GAD-7) [36] is a measure to screen for GAD and assess severity of GAD symptoms. The scale consists of 7 items, each measured on a Likert-type scale ranging from 0 (“not at all”) to 3 (“nearly every day”), and total score is obtained by summing all items. For each item, respondents are asked to consider how they have been feeling over the past 2 weeks. Anxiety symptom severity is categorized as: minimal (total score=0-4), mild (total score=5-9), moderate (total score=10-14), and severe (total score=15 or higher). The scale has acceptable internal reliability and good psychometric properties, including among general population samples [37].

The Patient Health Questionnaire-9 (PHQ-9) [38] is a measure to assess the severity of depression symptoms. The scale consists of 9 items, each measured on a Likert-type scale ranging from 0 (“not at all”) to 3 (“nearly every day”), and total score is obtained by summing all items. For each item, respondents are asked to consider how they have been feeling over the past 2 weeks. Depression symptom severity is categorized as: minimal (total score=0-4), mild (total score=5-9), moderate (total score=10-14), moderately severe (total score=15-19), and severe (total score=20 or higher). The scale has acceptable internal reliability (α=.86-.89) and overall sound psychometric properties across settings [39].

The Posttraumatic Stress Disorder Checklist (PCL-5) [40] is a measure to assess symptoms of PTSD. The scale consists of 20 items, each measured on a Likert-type scale ranging from 0 (“not at all”) to 4 (“extremely”), and total score is obtained by summing all items. In COVID Coach, the PCL-5 is administered with only a brief introduction, followed by the assessment items. For each item, respondents are asked to consider how they have been feeling over the past month. Initial research suggests that total scores of 31 to 33 (or higher) are indicative of probable PTSD. For this study, we use 33 as the cut-off for significant PTSD symptoms. The PCL-5 was found to be reliable and valid in both veteran [41] and civilian populations [42].

### Analyses

SQLPro Studio (Hankinsoft Development, Inc) was used for all data preprocessing and extraction. SAS University Edition (SAS Institute) software in conjunction with Oracle’s VirtualBox were used for all data analyses. We calculated descriptive statistics for key content usage, retention, and baseline levels of mental health symptom severity and levels of well-being. Chi-square analyses were conducted to understand differences in returning to the app for a second day of use based on key content usage on the first day of app use and baseline mental health symptoms. We ran separate chi-square analyses for each predictor. Independent samples *t* tests were conducted to examine differences in total unique days of app use and total manage stress tools utilized among app users who completed an assessment on their first day of app use compared to those who did not. An analysis of variance (ANOVA) was conducted with a Tukey test for post hoc analysis to examine differences in baseline WEMWBS scores, by month, among users who completed a well-being assessment on their first day of app use. Regression analyses were conducted to examine the relationship between baseline mental health symptoms and unique days of app usage.

## Results

### Reach and Reception

The app was released at the end of April 2020, and as of October 31, 2020, it has been downloaded 143,097 times. It is highly rated on both the Apple App Store (4.8 out 5 stars) and the Google Play Store (4.7 out of 5 stars). Users had the opportunity to provide written reviews along with star ratings. The majority of written reviews were overwhelmingly positive, with comments such as “Beautifully calming…,” “a necessity for our new normal,” “one of the best free apps I’ve found,” and “this is amazing… it has all you may need… mood trackers, resources, meditation-not too frilly, just important.” Notably, due to Google’s restrictions on mobile apps related to COVID-19 (including hiding certain results for apps among searches containing “COVID”), COVID Coach has been installed at a ratio of over 3:1 for iOS compared to Android mobile devices.

### Daily and Monthly Active Users

**Figure 1:**
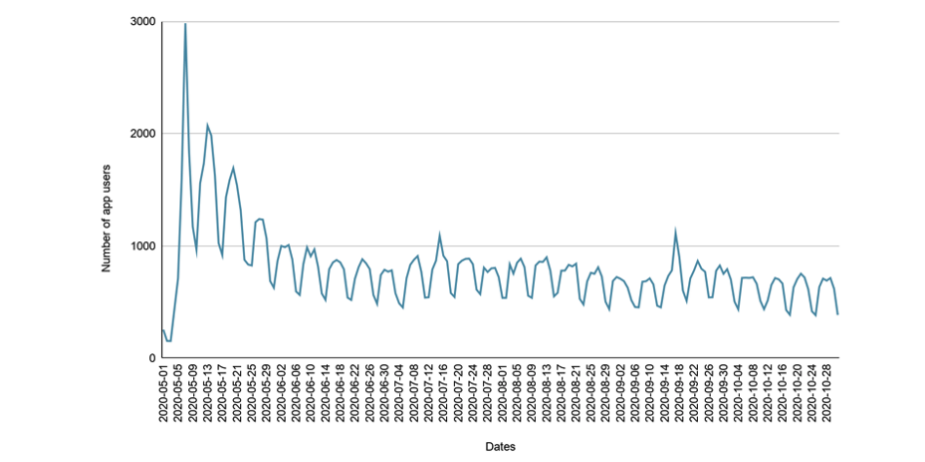
Daily active COVID Coach users, from May 1, 2020, through October 31, 2020.

The number of daily active users spiked in May 2020 (mean 1205.77, SD 615.70), shortly after the app’s release. The number of daily active users has leveled off but remained stable with average daily active users of 778.67 (SD 161.16), 752.03 (SD 152.07), 712.71 (SD 141.85), 682.83 (SD 150.83), and 611.35 (SD 128.60), respectively, during the months of June, July, August, September, and October 2020 (Figure 1). Although timestamp information is only captured in UTC, there appears to be a consistent, weekly pattern of usage such that the app is used more during the week than on weekends. The number of monthly active users followed a similar pattern as daily active users. The number of monthly active users peaked in May but remained steady through October 2020, at approximately 11,000 unique app users per month.

### Key Content Usage

Within the observation window (May 1, 2020, through October 31, 2020), there were 49,297 unique app users and 462,651 app events associated with the four key content areas (*Manage Stress*, *Learn*, *Mood Check*, and *Find Resources*). Table 1 provides an overview.

Of the four key sections of the app, the *Manage Stress* section, which contains tools for coping with stress and anxiety, was the most utilized. Across the observation window, there were 325,691 total tool use events (70.4% of all key events), among 28,009 unique install codes (56.82% of all unique install codes). Within the *Manage Stress* section, app users can directly select individual tools from a list of all tools, or they can have a tool recommended to them by selecting from one of seven possible challenges related to the pandemic: (1) coping with stress, (2) feeling lonely, (3) creating space for myself, (4) feeling sad or hopeless, (5) handling anger and irritability, (6) navigating relationships, and (7) sleep struggles. Across all app users, 48.5% (n=23,885) selected at least one challenge. Among this group of app users, challenges related to coping with stress were the most commonly selected (n=12,696, 53.2%), followed by sleep struggles (n=9308, 39.0%) and feeling lonely (n=9153, 38.3%).

**Table 1:** Overall key content usage among all COVID Coach users within the observation window (between May 1, 2020, and October 31, 2020).

Key content area (specific in-app action)	Unique app users, n (%)^a^	Key events, n (%)^b^	Totals per app user, mean (SD); range
Manage Stress (tried at least one tool)	28,009 (56.8)	325,691 (70.4)	11.63 (30.33); 1-2124
Learn (viewed at least one topic)	10,124 (20.5)	52,123 (11.3)	5.15 (8.09); 1-267
Mood Check (entered and rated at least one goal or completed at least one assessment )	13,510 (27.4)	47,821 (10.3)	3.54 (12.52); 1-1008
Find Resources (viewed at least one specific subsection)	9418 (19.1)	37,016 (8.0)	3.93 (7.82); 1-329

^a^Total number of unique app users during the observation window=49,297. Percentage of total app users. Percentages in this column will not sum to 100% because app users could have completed actions across the four types of key content areas.

^b^Total key app events during the observation window=462,651. Percentage of total key app events.

Overall, the psychoeducation content within the *Learn* section of the app was consumed less frequently and by fewer users than the *Manage Stress* tools. Within the observation sample, there were 52,123 unique learn topic views (11.3% of all key events), among 20.5% of all app users (10,124/49,297). Four out of the five most viewed topics appeared in the first subsection within *Learn* (Staying Well).

In total, core activities within the *Mood Check* section comprised 10.3% (47,821/462,651) of all key events. Across the observation window, 27.4% of all app users (13,510/49,297) submitted at least one goal success rating or completed at least one of the four available assessments in the *Mood Check* section. There were 10,253 submitted goal success ratings across 2285 app users (4.6% of the total sample), and 37,568 completed assessments across 13,223 unique app users (26.8% of all users).

Across the eleven subsections within *Find Resources*, 19.1% (9418/49,297) of all app users viewed the resource pages 37,016 times across the observation window, representing 8% of all key events. Notably, although not the most frequently viewed subsection, *Crisis Resources* (which includes direct links to phone lines, text support, and online chat for services such as the National Suicide Prevention Lifeline, Crisis Text Line, and Substance Abuse and Mental Health Services Administration’s Helpline) was visited 3297 times (8.9% of all *Find Resources* visits) among 2131 unique users. Table 2 presents detailed information about key events within each of the four key sections.

**Table 2:** Detailed key content usage among all COVID Coach users within the observation window (between May 1, 2020, and October 31, 2020).

Key content area		Unique app users, n (%)^a^	Key app events, n (%)^b^
**Manage Stress: top 5 most frequently used tools**			
	Ambient Sounds (an audio-only tool with no narration)	7041 (14.3)	18,493 (4.0)
	Deep Breathing (an audio-guided exercise)	7870 (16.0)	16,011 (3.5)
	Change Your Perspective (a tool with tips for how to replace negative thoughts with more helpful ones)	6721 (13.6)	12,480 (2.7)
	Muscle Relaxation (an audio-guided exercise focused on relaxing distinct core body parts)	6037 (12.2)	11,599 (2.5)
	Grounding (a tool with tips on how to stay connected to the present moment and surroundings)	5718 (11.6)	9767 (2.1)
**Learn: top 5 most frequently viewed topics**			
	Prioritizing Yourself, Right Now	2131 (4.3)	2811 (0.6)
	Managing Irritability	1856 (3.8)	2281 (0.5)
	Finding Humor	1506 (3.1)	1817 (0.4)
	Finding Calm	1442 (2.9)	1813 (0.4)
	Sleep	1184 (2.4)	1511 (0.3)
**Find Resources: top 3 most frequently viewed sections**			
	Finding Local Resources (for locating state-specific COVID-19 guidelines and information)	2927 (5.9)	6451 (1.4)
	Meeting Your Needs (for basic needs support)	3176 (6.4)	6223 (1.3)
	Mobile Apps to Support Mental Health (information about other free apps to support mental health)	2461 (5.0)	3748 (0.8)
**Mood Check: completion of assessments, by type**			
	Track Mood (PHQ-9^c^)	7698 (15.6)	11,732 (2.5)
	Track Anxiety (GAD-7^d^)	8115 (16.5)	11,649 (2.5)
	Track Well-Being (WEMWBS^e^)	6151 (12.5)	8860 (1.9)
	Track PTSD^f^ Symptoms (PCL-5^g^)	3568 (7.2)	5327 (1.2)

^a^Total number of unique app users during the observation window=49,297. Percentage of total app users.

^b^Total key app events during the observation window=462,651. Percentage of total key app events.

^c^PHQ-9: Patient Health Questionnaire-9.

^d^GAD-7: Generalized Anxiety Disorder-7.

^e^WEMWBS: Warwick-Edinburgh Mental Well-Being Scale.

^f^PTSD: posttraumatic stress disorder.

^g^PCL-5: Posttraumatic Stress Disorder Checklist-5.

### Return Usage and Retention

Among the 49,297 app user install codes present in the observation window, 86.8% (n=42,783) of COVID Coach users had their first day of app use occur within the observation window. Thus, for the analyses presented in this section, usage patterns will be restricted to only the app users and events associated with those whose first day of app use occurred during the observation window.

Nearly half of COVID Coach users used the app for a single day (n=20,793, 48.6%), and an additional 32.3% (n=13,818) used the app for 2 or 3 days in total. Less than 2% of the sample (n=709) used the app for 15 or more distinct days (Table 3). On average, across all app users with ≥2 distinct days of app use (n=21,990), the number of days retained was 42.44 (SD 44.40, median 25, range 1-179). On average, the number of days between the first day of app use and the second day of app use was 14.65 (SD 24.52, median 4, range 1-176). Although the majority of app users who returned to the app for at least a second day returned within 14 days, there was variability, including users whose second day of use occurred over 90 days after the first (see Table 4 for a detailed analysis among users whose first month of use occurred in May, June, or July so that returns within a 90-day or longer window could be examined).

**Table 3:** Total number of distinct days of COVID Coach use, by month of first app use.

Month of first app use	Frequency of users per distinct day, n (%)					
	1 day only	2 days	3 days	4-6 days	7-14 days	≥15 days
May	6573 (42.67)	3406 (22.11)	1891 (12.28)	2137 (13.87)	1049 (6.81)	348 (2.26)
June	2533 (44.26)	1205 (21.10)	693 (12.14)	770 (13.49)	372 (6.51)	137 (2.40)
July	2939 (48.79)	1246 (20.68)	640 (10.62)	737 (12.23)	362 (6.01)	100 (1.66)
August	3165 (51.31)	1267 (20.54)	648 (10.51)	698 (11.32)	314 (5.09)	76 (1.23)
September	2783 (53.66)	1119 (21.58)	548 (10.57)	503 (9.70)	190 (3.66)	43 (0.83)
October	2800 (65.25)	849 (19.79)	306 (7.13)	273 (6.36)	58 (1.35)	5 (0.12)
All	20,793 (48.6)	9092 (21.25)	4726 (11.05)	5118 (11.96)	2345 (5.48)	709 (1.66)

**Table 4:** Analysis of days between first and second app use among COVID Coach users with at least 2 distinct days of app use, by month of first use.

First month of app use	Users that returned at least once, n	First return within 7 days, n (%)	First return within 8-14 days, n (%)	First return within 15-30 days, n (%)	First return within 31-60 days, n (%)	First return within 61-90 days, n (%)	First return after more than 90 days, n (%)
May	8831	4956 (56.12)	1071 (12.13)	1107 (12.54)	870 (9.85)	391 (4.43)	436 (4.94)
June	3177	1786 (56)	403 (12.68)	457 (14.38)	298 (9.38)	118 (3.71)	115 (3.62)
July	3085	1922 (62.30)	385 (12.48)	366 (11.86)	232 (7.52)	130 (4.21)	50 (1.62)

#### Differential Day 2 Return Rates Based on Day 1 Key Content Usage

On both the first and second days of app use (see Table 5 for an overview of usage), many app users tried at least one tool within the *Manage Stress* section (46.80% [n=20,222] on the first day, 41.85% [n=9202] among individuals who returned to the app for a second day). Usage of the key content in COVID Coach predicted returning to the app for a second day.

Of those who tried at least one *Manage Stress* tool on their first day of app use, 57.2% (n=11,444) returned for a second visit; whereas only 46.3% (n=10,546) of those who did not try a tool returned (*P*<.001). Among those who viewed at least one *Learn* topic on their first day of app use, 58.8% (n=3292) returned for a second day of use; whereas only 50.3% (n=18,698) who did not view a learn topic returned (*P*<.001). With respect to the *Mood Check* section, 57.2% (n=4892) of app users that completed at least one goal rating or one assessment activity returned for a second day of use, compared to 50.0% (n=17,098) of users who did not complete any *Mood Check* activities (*P*<.001). Lastly, among app users who viewed at least one specific *Find Resources* subsection, 57.4% (n=3014) returned for a second day of app use, compared to only 50.6% (n=18,976) of users who did not view any resources returned (*P*<.001).

Additionally, usage patterns among individuals who completed an assessment on the first day of app use were significantly different than those who did not complete an assessment on their first day. On average, individuals who completed at least one assessment on their first day of app use utilized COVID Coach for more unique days within the observation window (mean 3.29 days, SD 5.44) compared to individuals who did not complete an assessment on the first day (mean 2.66 days, SD 4.37; *P*<.001). Similarly, individuals who completed at least one assessment on their first day of app use utilized, on average, significantly more *Manage Stress* tools within the observation window (mean 9.2 tools, SD 24.6) compared to individuals who did not complete an assessment on the first day (mean 5.8 tools, SD 19.3; *P*<.001).

**Table 5:** Comparison of key content area usage, by first and second day of app use.

Number of key content areas accessed		App users	
		First day of app use (n=42,783), n (%)	Second day of app use (n=21,990), n (%)
**All four key areas**			
	Completed at least one action within all four key content areas	650 (1.5)	192 (0.9)
**Two to three key areas**			
	Manage Stress (with one or two other key areas; tried at least one tool and completed another action within one or two other key areas)	7953 (18.6)	3143 (14.3)
	Two or three key areas (excluding Manage Stress; completed at least one action within two or more of the Learn, Mood Check, or Find Resources sections)	1129 (2.6)	405 (1.8)
**One key area**			
	Manage Stress only (only tried at least one tool)	11,419 (26.7)	5867 (26.7)
	Mood Check only (only completed at least one goal rating or assessment)	2677 (6.3)	1355 (6.2)
	Find Resources only (only viewed at least one resource subsection)	1196 (2.8)	660 (3.0)
	Learn only (only viewed at least one learn topic)	805 (1.9)	485 (2.2)
**No key area actions**			
	Did not complete an action within any of the four key areas	16,954 (39.6)	9883 (44.9)

### Characterizing Baseline Mental Health Among COVID Coach Users

Baseline well-being among individuals using COVID Coach appeared to be relatively low and decreased over time. Among app users who completed a WEMWBS assessment on their first day of app use (n=3558, 8.32% of all users whose first day of app use occurred during the observation window), average well-being scores, by month, were all less than 42, which has been used as a cut-off to identify low well-being [34]. These average baseline scores decreased over time, with app users who completed their first WEMWBS on their first day of app use in September 2020 (n=416; mean 38.7, SD 0.04) or October 2020 (n=341; mean 38.1, SD 9.50) demonstrating significantly lower average well-being scores than app users who completed their first WEMWBS on their first day of using the app in May 2020 (n=1361; mean 41.2, SD 9.65).

Symptoms of anxiety, depression, and PTSD were prevalent among app users. For all app users who completed a GAD-7 assessment on their first day of app use (n=4870; 11.4% of users), 12.8% (n=625) had scores suggesting minimal anxiety (total score=0-4), 32.1% (n=1565) endorsed mild levels of anxiety (total score=5-9), 25.2% (n=1225) indicated moderate levels of anxiety (total score=10-14), and 29.9% (n=1455) of scores fell into the severe symptom range (total score=15 or higher).

Among app users who completed a PHQ-9 on their first day of app use (n=4548, 10.6% of users), 16.5% (n=749) had scores suggesting minimal depression (total score=0-4), 28.9% (n=1312) endorsed mild levels of depression (total score=5-9), 25.0% (n=1136) indicated moderate levels of depression (total score=10-14), 17.5% (n=795) endorsed moderately severe levels of depression (total score=15-19), and 12.2% (n=556) of scores fell into the severe symptom range (total score=20 or higher).

Unlike the GAD-7 and the PHQ-9, the PCL-5 does not have symptom severity categorizations. However, among app users who completed a PCL-5 on their first day of app use (n=2064, 4.8% of users), the majority of individuals who completed the assessment (n=1234, 59.8%) had a total score ≥33, which is consistent with significant PTSD symptoms. 

### Baseline Mental Health Characteristics and Return Usage

Baseline PTSD symptoms predicted returning to the app for a second day. Among individuals with a baseline PCL-5 score of 33 or greater, 62.5% returned to the app for a second day of use, compared to only 56.4% of individuals with scores below 33 (*P=*.006). Neither symptom severity for anxiety or depression nor levels of well-being were predictive of return usage.

We conducted regression analyses to examine the relationship between baseline mental health symptoms and unique days of app usage. Depression and PTSD symptoms were predictive of the total number of unique days of app use. With respect to depression symptoms, we utilized the group with minimal symptoms as the reference group in comparison to those with mild, moderate, moderately severe, and severe symptoms. On average, those with moderate levels of depression on their first day of app use returned to the app for a greater number of days (mean 3.72 days) than those with minimal symptoms of depression (mean 3.08 days; *t*_1_=3.01, *P*=.003). Individuals with mild, moderately severe, and severe depression did not significantly differ from the reference group. Although the difference in usage between moderately severe and minimal symptom severity categories was not statistically significant, it was trending in the predicted direction. With respect to PTSD symptoms, individuals with baseline PCL-5 scores indicating significant PTSD symptoms utilized the app for a significantly greater number of days (mean 3.79 days) than those with subthreshold symptom levels (mean 3.13 days; *t*_1_=2.29, *P=*.02).

## Discussion

### Principal Findings

This exploration of COVID Coach usage among the general population suggests that mobile apps may have the reach and accessibility necessary to be a useful medium for disseminating mental health information and resources to individuals experiencing stress related to the COVID-19 pandemic.

Between May 1, 2020, and October 31, 2020, the app was used by nearly 50,000 individuals, and daily active usage has remained steady over time. In addition to the total number of individuals reached, the key content within the app was utilized in over 450,000 instances. The stress management tools were most frequently used with over 28,000 users utilizing individual tools over 300,000 times. Further, each of the other three key content areas in the app were accessed tens of thousands of times by tens of thousands of users. This reach and scalability of COVID Coach across the general population is an example of how digital mental health tools can become successfully integrated into disaster response strategies. From a public mental health perspective (eg, [43]), being able to rapidly deploy evidence-informed tools and reliable health information via a free, accessible, and secure app is a way for the federal government to contribute to a digital mental health safety net and reduce barriers to accessing mental health resources.

Importantly, COVID Coach appears to be reaching individuals in need of mental health resources. On average, among app users who completed assessments during their first day of use, well-being was low, and the majority of individuals were indicating greater than minimal symptoms of anxiety, depression, and PTSD. Additionally, among app users who identified challenges they are facing, the majority reported difficulties with managing stress, troubles with sleep, and feelings of loneliness. We cannot determine if individuals utilizing COVID Coach are representative of the general population, but elevated levels of anxiety, depression, and posttraumatic stress are consistent with other research conducted during the pandemic [6,7,44]. Individuals with significant PTSD symptoms at baseline were more likely to return to the app for a second day of app use. On average, individuals with significant PTSD symptoms used the app for a greater number of days than those with subthreshold symptoms, and individuals with moderate depression used the app for more days than those with minimal symptoms. Greater usage among individuals with moderate depression symptoms is consistent with previous research [45].

Although overall app utilization data suggested considerable reach, engagement proved to be less consistent. Our analyses revealed that the majority of COVID Coach users (80.9%) utilized the app on ≤3 days. This finding is consistent with research indicating that self-management apps for mental health are often not used over extended periods of time [26,27,46]. However, as noted by Ng and colleagues [47], there is a need for more standardized reporting of measures related to user engagement and retention. The average number of retention days, as well as the number of days between days of use, suggest that the app may not be something that individuals use on a daily basis, but rather during moments of distress or need. This type of usage is consistent with the overall design of COVID Coach as a self-management tool, which does not provide any guidance on how often or when to use the app.

This research also provides some guidance on how engagement might be encouraged in future app versions. In general, app users that completed actions within the key content areas on the first day of app use were more likely to return for a second day of app use. More specifically, users that completed an assessment on the first day of app use were significantly more likely to use the app for a greater number of days and to use a greater number of stress management tools than app users who did not complete an assessment on the first day of app use. These findings suggest that finding ways to motivate users to complete actions within key areas on their first day of app use, particularly tools and assessments, may be one way to enhance engagement and retention. For example, having recommendations for a tool or assessment to try, easily accessible from the app home screen, may encourage users to try a specific in-app activity. Additionally, the onboarding sequence could include a few brief questions to help tailor in-app recommendations to the user’s intentions and preferences, and guide them through the process of setting customized goals for using the features within the app most relevant to them. Lastly, finding ways to regularly disseminate and highlight new app content (eg, managing stress around prolonged distance learning, vaccine information) may encourage users to return to the app more frequently.

### Limitations

Because COVID Coach does not collect any identifying information, we cannot say anything about the populations that we have reached, other than what we can characterize based upon in-app actions. Future research that permits collection of identifying information is needed, particularly given the disproportionate impact the pandemic has had on vulnerable groups of people. A Spanish version of COVID Coach has recently been released, and plans for data collection on app usage within Spanish-speaking populations are underway.

Additionally, we utilize the unique install codes as a proxy for an individual user. We assume that most individuals do not delete and reinstall the app multiple times. However, if an individual were to download COVID Coach on more than one mobile device, or delete it and reinstall, each of those installations would be assigned a unique install code, and would appear as a new user.

Although the app includes assessments for individuals to self-monitor well-being and symptoms of anxiety, depression, and PTSD, it is difficult to reliably measure change in these constructs via the app, due to the naturalistic nature of this study and the changing landscape of the pandemic over time. It is important to highlight that even though a score of 33 or higher on the PCL-5 is suggestive of PTSD, the assessment questions in the app do not ask app users to respond to the questions while focusing on a particular traumatic incident, so caution in interpreting the meaning of these scores is warranted. Because the PCL-5 refers to “the stressful experience” in each item, in the context of COVID Coach, the PCL-5 may be capturing overall levels of distress. While desirable, we also did not have a way to measure other potential proxy variables of interest such as coping self-efficacy, perceived helpfulness of the app, improved opinions about mental health care, or reduction in stress related to enhanced support access, as these cannot be determined solely by in-app usage data.

Future research is needed to better understand who is interested in public mental health apps like COVID Coach, what their primary goals are for using the app, which outcomes are most useful in understanding engagement patterns, and how successful usage is defined. For example, someone may use the app only once, find the exact resource they need, and not use the app again, whereas someone else may be experiencing significant stress, use tools in moments of distress, and track mental health symptoms on a weekly basis. Findings from this type of research could be used to advance the science of mobile mental health and also be directly applied to a suite of publicly available apps that have been downloaded over 4 million times and are in widespread use across the VA, the largest health care organization in the United States.

### Conclusions

As the mental health impacts of the pandemic continue to be widespread and increasing, digital health resources, such as apps like COVID Coach, are a scalable way to provide evidence-informed tools and resources. We believe that this is the first evaluation of a mobile mental health app designed specifically for use during the COVID-19 pandemic. This work shows that tens of thousands of people are accessing the app, with a particular focus on the tools for stress and coping. Such rapid uptake of a public mobile mental health app is unprecedented and signals perceived value. Specially, the findings from this evaluation suggest that apps may play a helpful role in providing mental health resources in the context of a public health disaster.

Future research should attempt to elucidate for whom and under what conditions the app is most helpful, and how to increase and sustain engagement. Additional areas of focus should include how to optimize the app for populations impacted by disparities related to mental health literacy, digital literacy, and stigma around mental health care. As noted by many mHealth (mobile health) scholars [48-50], there is no reason to believe that digital mental health care and blended options will disappear after the pandemic, so it is important to find strategies for increasing reach and optimizing for engagement within self-management tools. These strategies must also attend to issues of health inequities [48,49,51]. Due to the scale of the crisis, the pandemic may have opened the door to conversations about mental health, and apps may be a helpful first step in providing tools, accurate information, and connecting people with reliable resources. Those in government and nonprofit organizations may be able to provide these kinds of tools as a way to contribute to a digital mental health safety net and help alleviate mental health disparities.

## Appendix

Multimedia Appendix 1 COVID Coach screenshots.

## Abbreviations

ANOVA: analysis of variance
BIPOC: Black, Indigenous, people of color
GAD-7: Generalized Anxiety Disorder-7
JSON: JavaScript Object Notation
mHealth: mobile health
PCL-5: Posttraumatic Stress Disorder Checklist-5
PHQ-9: Patient Health Questionnaire-9
PTSD: posttraumatic stress disorder
UTC: Coordinated Universal Time
VA: Veterans Affairs
WEMWBS: Warwick-Edinburgh Mental Well-Being Scale
