# Supplementary material for: Exploring Usage of COVID Coach, a Public Mental Health App Designed for the COVID-19 Pandemic: Evaluation of Analytics Data
Source: J Med Internet Res. 2021 Mar 1;23(3):e26559. doi: 10.2196/26559 (PMC7924218; doi:10.2196/26559)

COVID Coach Home Screen

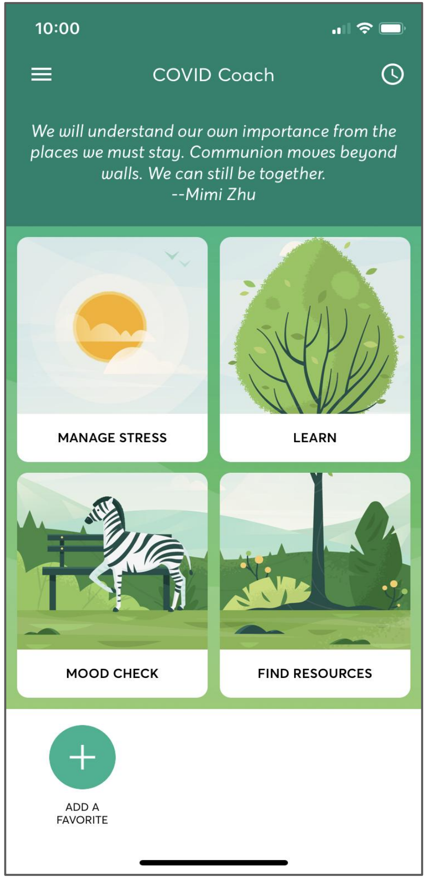

Manage Stress Overview

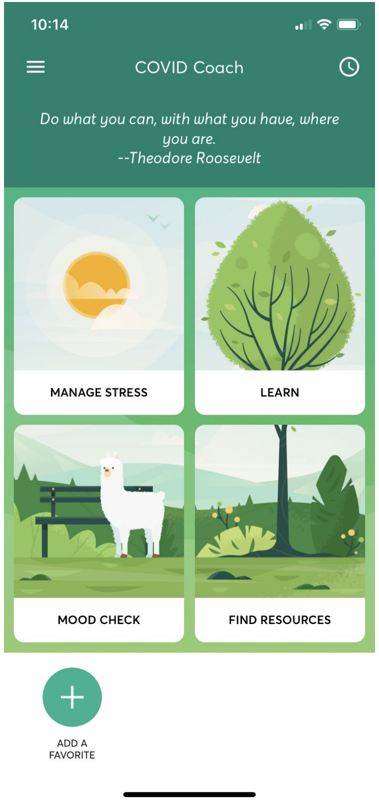

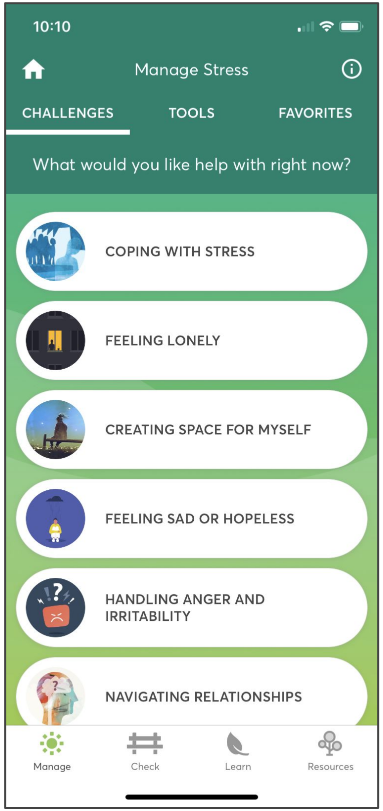

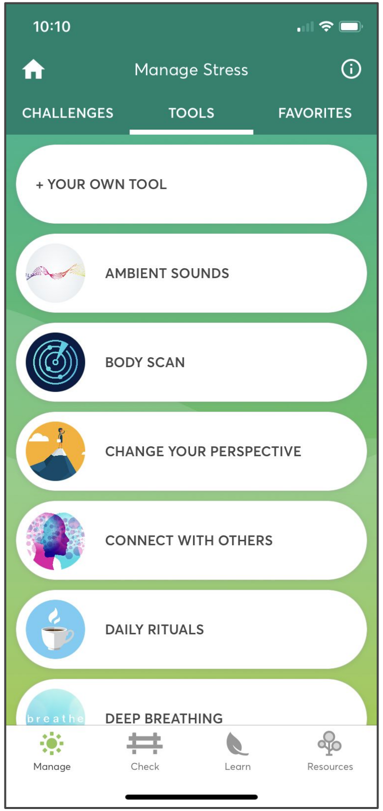

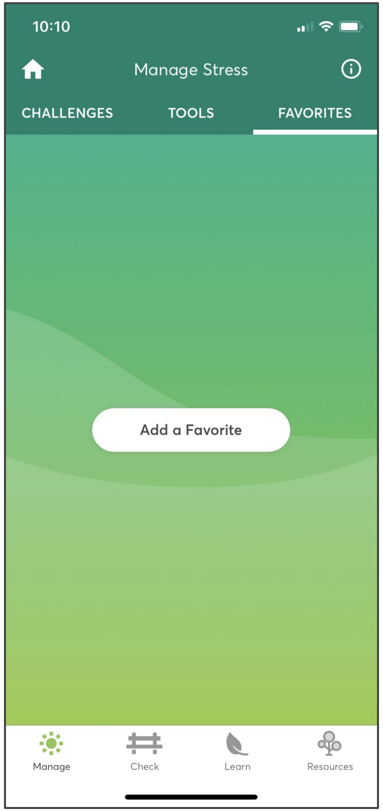

Manage Stress: Coping Tool Example 1

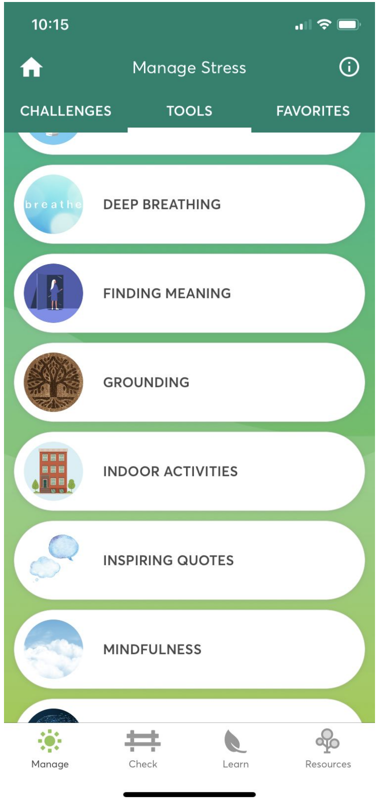

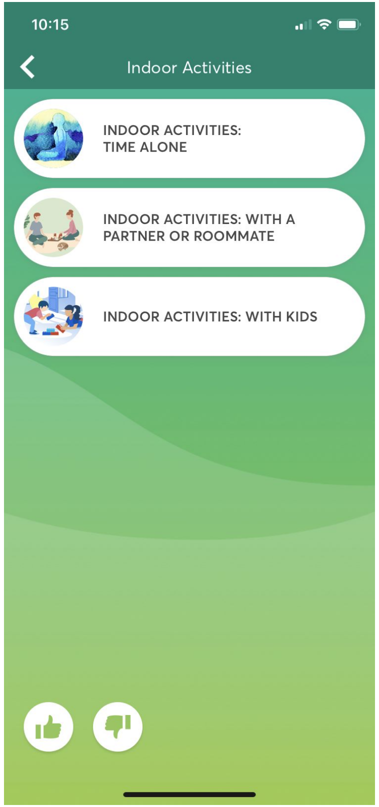

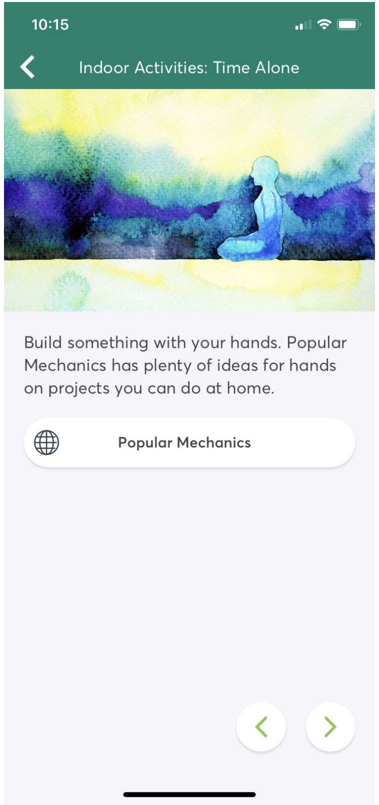

Manage Stress: Coping Tool Example 2

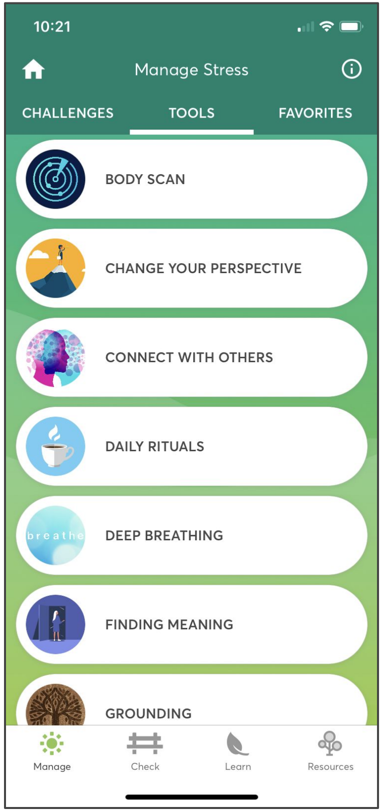

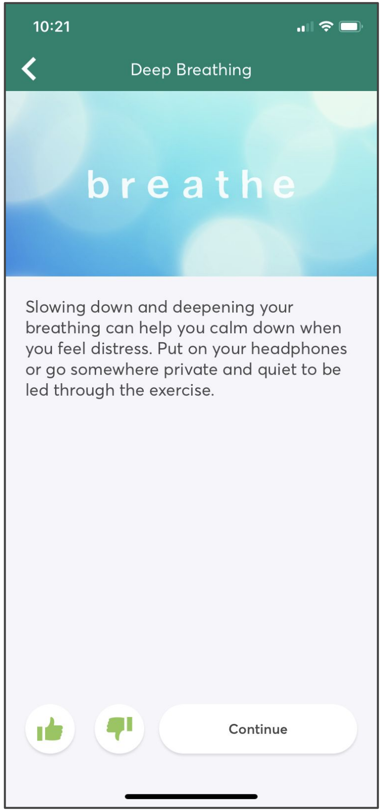

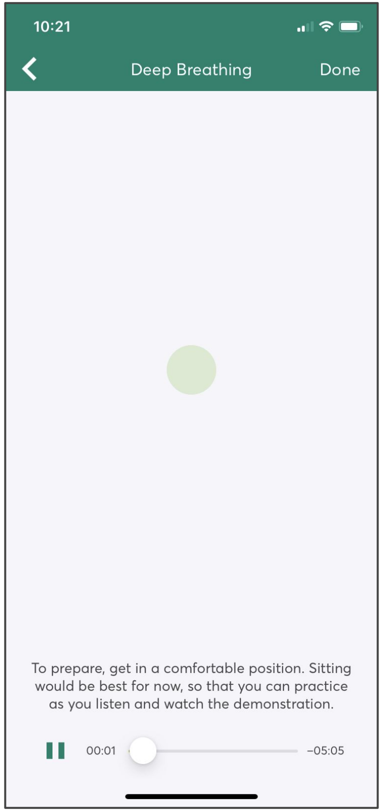

Learn Overview

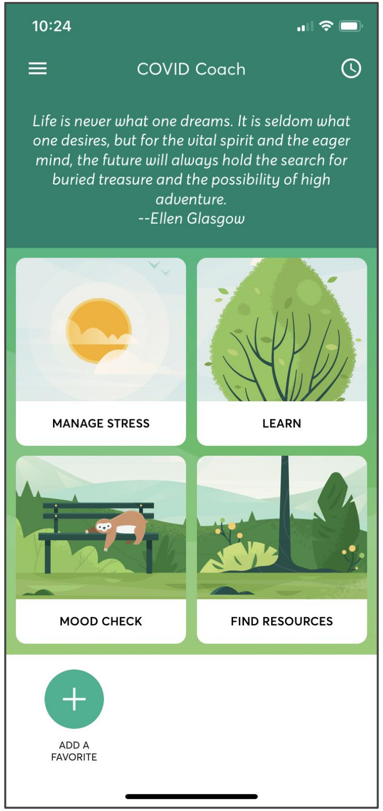

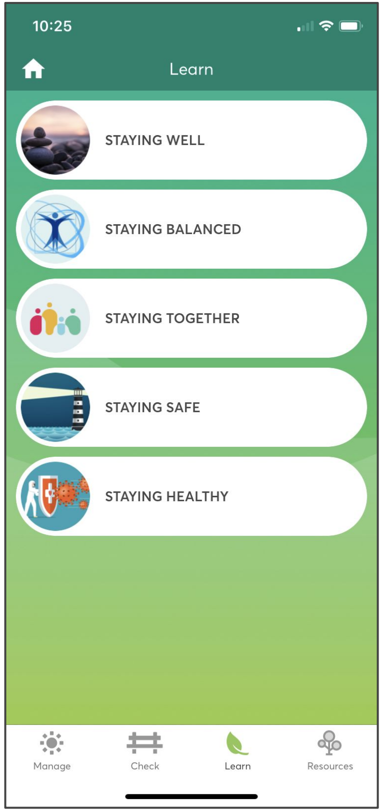

Learn > Sub-sections

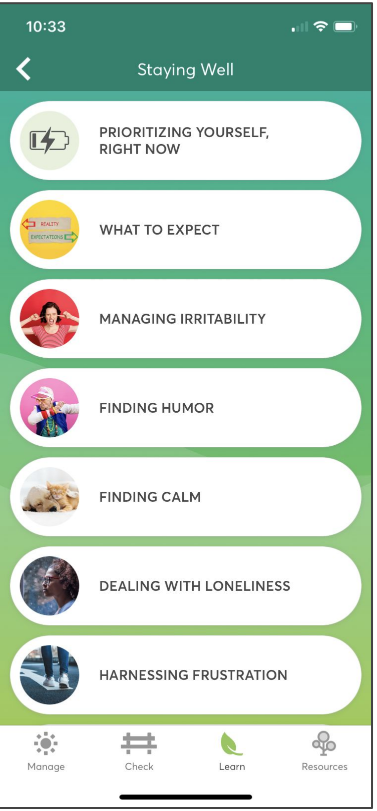

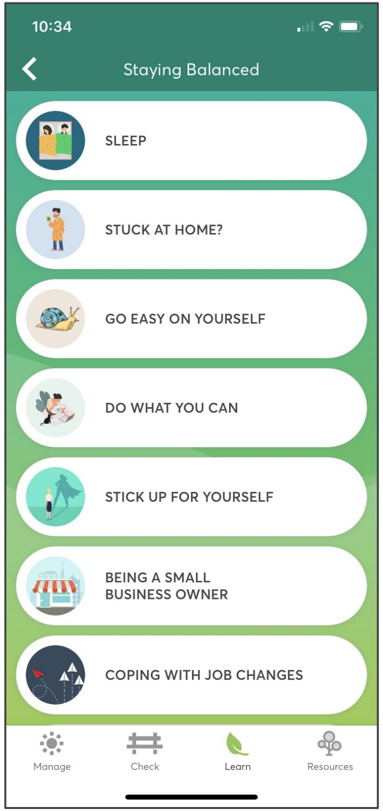

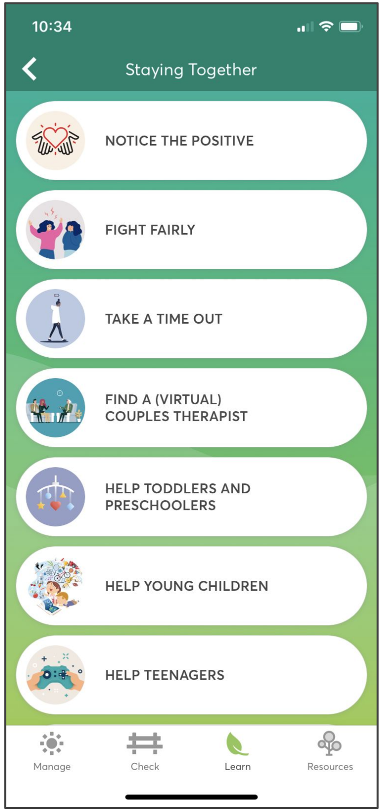

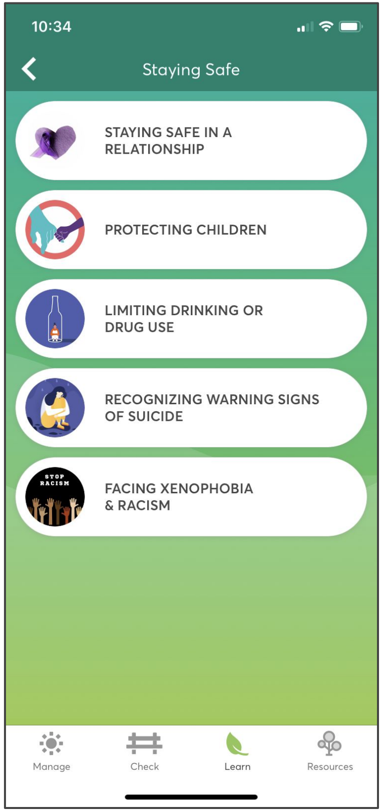

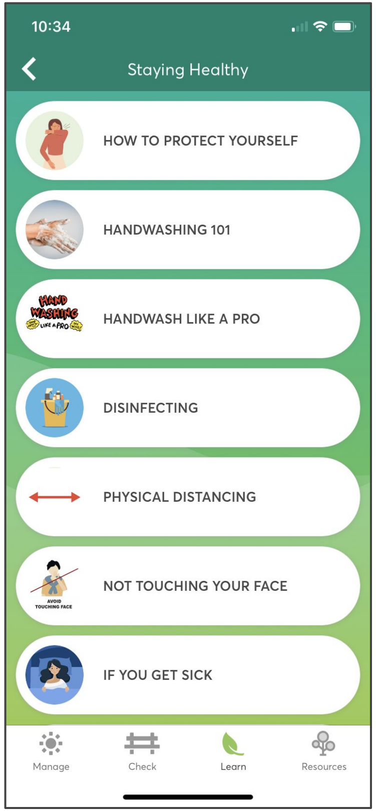

Mood Check Overview

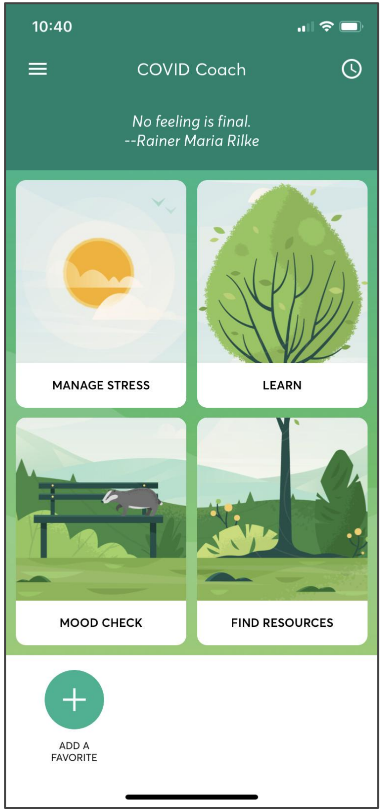

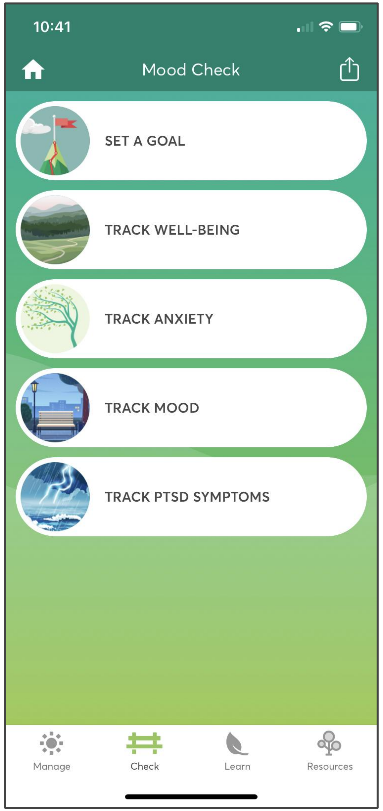

Mood Check: Set A Goal

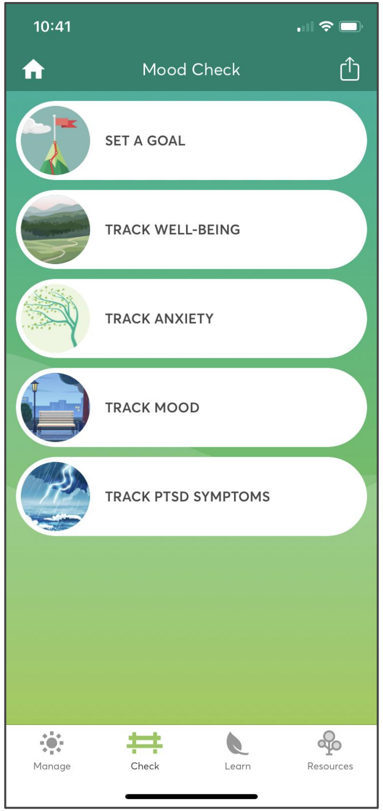

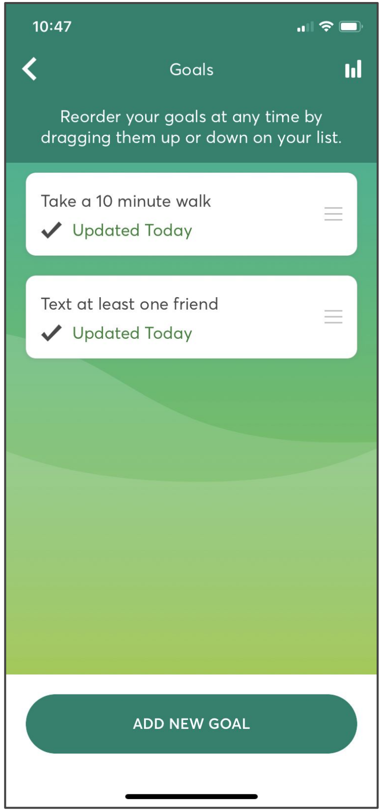

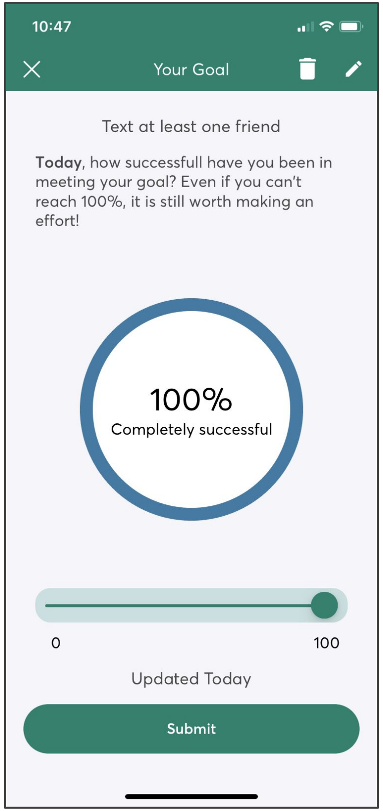

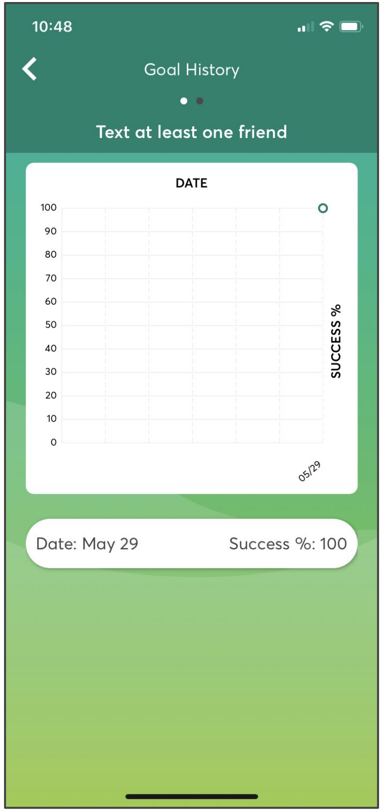

Mood Check: Track Well-Being (WEMBWS)

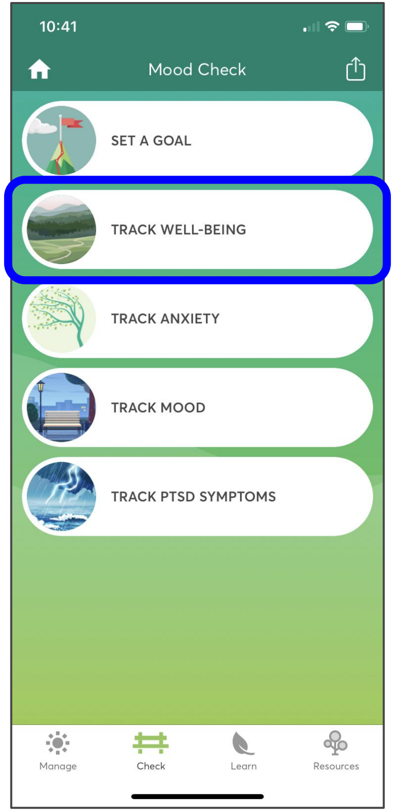

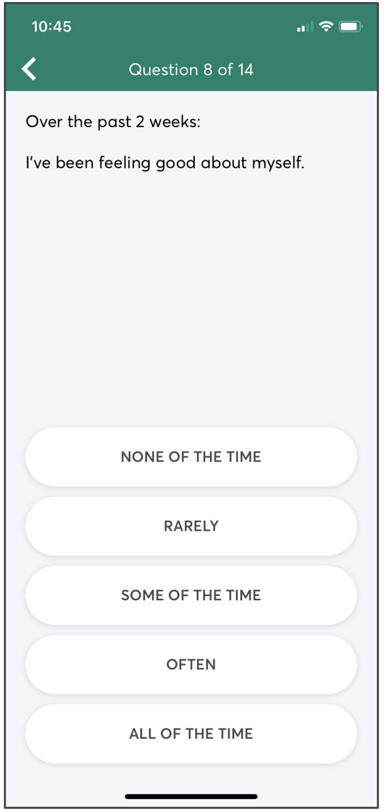

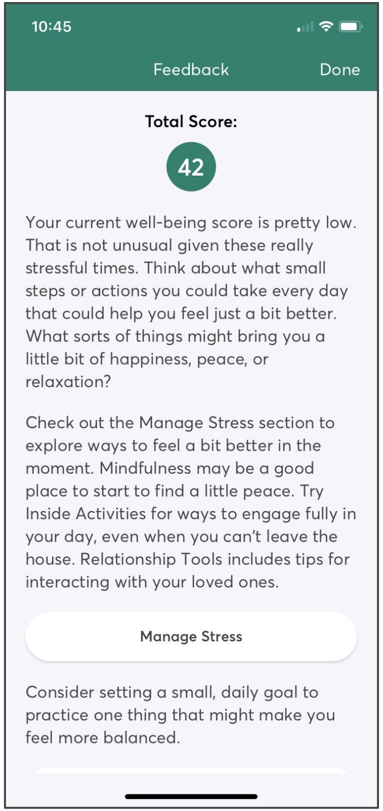

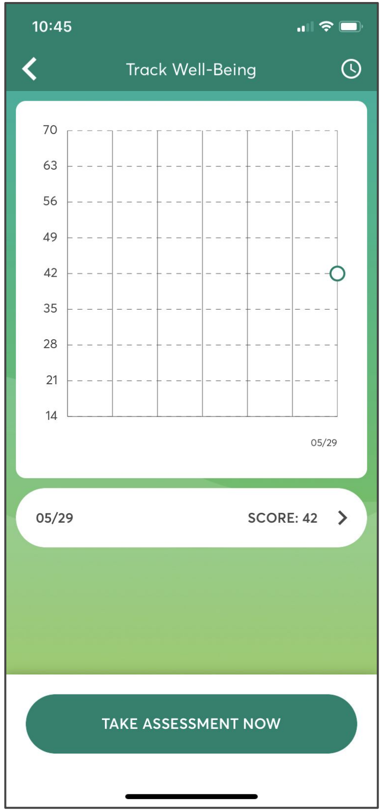

Find Resources Overview

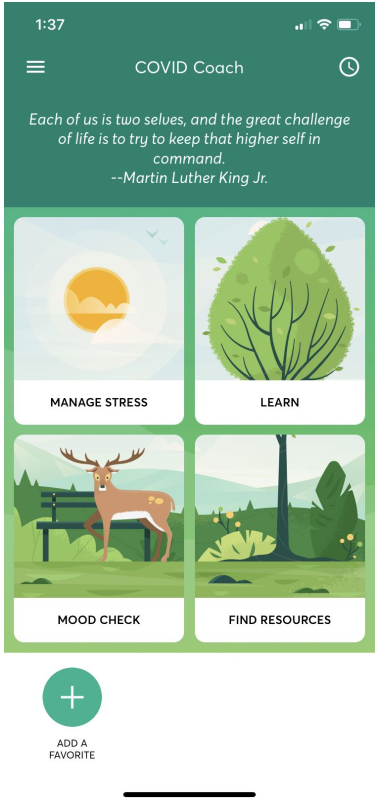

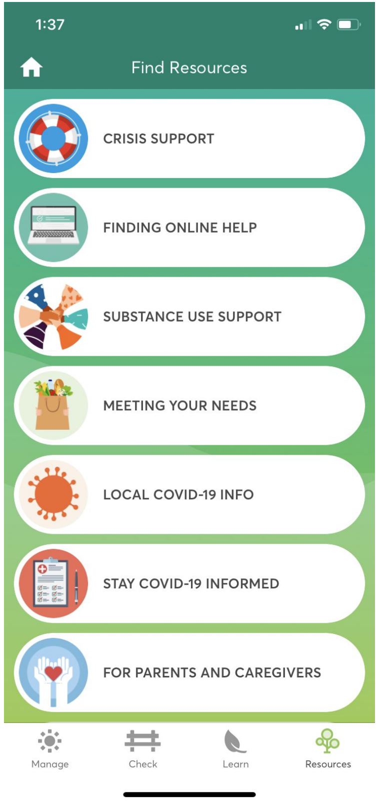

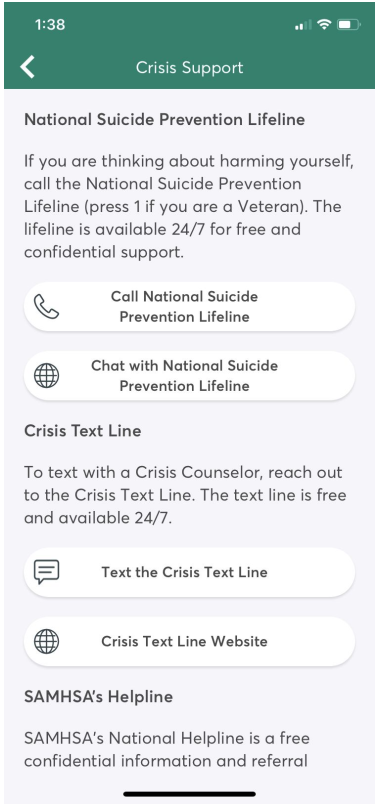

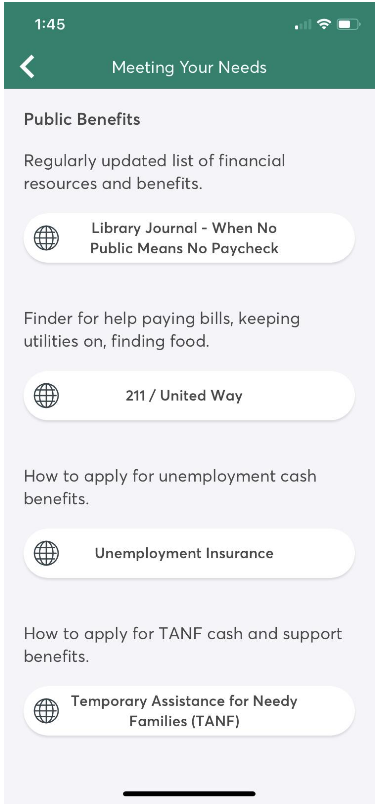

Supplement: Multimedia Appendix 1 [file jmir_v23i3e26559_app1.pdf]
